# A cross-sectional study explores the association of physical activity with the severity of peripheral arterial disease from the Hispanic Community Health Study/Study of Latinos (HCHS/SOL)

**DOI:** 10.1097/MD.0000000000032505

**Published:** 2022-12-30

**Authors:** Monira I. Aldhahi, Mohammed M. Alshehri, Abdulfattah S. Alqahtani

**Affiliations:** a Department of Rehabilitation Sciences, College of Health and Rehabilitation Sciences, Princess Nourah bint Abdulrahman University, Riyadh, Saudi Arabia; b Physical Therapy Department, College of Applied Medical Sciences, Jazan University, Jazan, Medical Research Center, Jazan University, Jazan, Saudi Arabia; c Department of Rehabilitation Sciences, College of Applied Medical Sciences, King Saud University, Riyadh, Saudi Arabia.

**Keywords:** ankle–brachial index (ABI), cardiovascular, moderate-to-vigorous physical activity (MVPA), peripheral arterial disease (PAD), physically inactive, severity

## Abstract

Engaging in physical activity (PA) has been proved to reduce the risk of developing cardiovascular diseases. In patients with peripheral arterial disease (PAD), diminished PA predicts high overall mortality. However, the extent of the association of participation in PA with PAD severity is unknown. Therefore, the overarching aim of this study was to investigate the association between PAD severity, PA levels and patterns using the Hispanic Community Health Study/Study of Latinos. This was a cross-sectional cohort study that included 495 participants with PAD and a total of 12,281 participants without PAD from the Hispanic Community Health Study/Study of Latinos database. The Global Physical Activity Questionnaire was administered to assess the time spent weekly in performing moderate-to-vigorous PA (MVPA) during work, leisure time, and transportation. The ankle–brachial index (ABI) was used to measure PAD. PA status was categorized on the basis of MVPA as follows: physically active and physically inactive to insufficient. In addition, all participants were classified as follows: those with normal ABI who were physically active, those with normal ABI but who were physically inactive, those with PAD but were physically active, and those with PAD who were physically inactive. Complex sample for regression models were used to investigate the association between PA and the severity of PAD. Of the participants, 235 (47.5%) were physically inactive to insufficient, and 260 participants (52.5%) engaged in at least 150 min/wk of MVPA, which is the recommended PA level according to the guidelines of World Health Organization. Compared with who were highly active, the participants who engaged in low PA were twice as likely to have moderately severe ABI and 4 times as likely to have severe ABI, after adjustment for the covariates (age, smoking status, and body mass index). Hispanic/Latino adults with sever PAD in the US showed pattern of physical inactivity. Findings of this study highlight the association between PA and severity of PAD. These findings highlight the necessity of interventions in increasing PA in these participants. Future studies are required to identify appropriate exercise regimens or home-based programs to help patients with severe PAD meet the current PA recommendations.

## 1. Introduction

Peripheral arterial disease (PAD) is prevalent in nearly 8.5 million people in the US, and the number of concerns is rising, with PAD increasing in those who have diabetes, hypertension, and hypercholesterolemia.^[1]^ In addition, a sedentary lifestyle is a risk factor for developing PAD.^[1]^ In the Hispanic population, the age-adjusted death rates for PAD were 13.4 and 9.1 for men and women, respectively.^[1]^ The ankle–brachial index (ABI) is an indicator of the systolic pressure ratio measured in the ankle and arm, and it is used to diagnose and assess PAD severity.^[2]^ ABI values between 1.0 and 1.4 are considered normal, whereas values <1.0 or >1.4 are considered abnormal.^[3–5]^ The hazard ratios of deaths in men and women due to cardiovascular reasons were higher in people who had ABI values <1.0 or >1.4, which makes the ABI a suitable predictor of cardiovascular mortality or events. In people with ABI values <1, the cardiovascular mortality rates in men and women were 18.7% and 12.6%, respectively.^[1,2]^ Thus, clinicians should pay attention to those who have abnormal ABI values and try to find appropriate interventions to minimize the risks of abnormal ABI.

Exercise, either supervised or home-based, is beneficial in reducing the unfavorable symptoms of PAD and increasing the distance of symptom-free walking in people diagnosed with PAD.^[1,5,6]^ Research has established a positive association between moderate-to-vigorous physical activity (MVPA) and health-related quality of life.^[1,5,6]^ A reduction of 9.5% in the ABI was reported as an immediate effect of 50 heel raise exercises.^[7]^ Less attention has been paid to physical activity (PA) levels in people with PAD. Gerage et al^[8]^ found that men with a mean age of 67 years and diagnosed with PAD spent a mean of 15 min/d doing MVPA, whereas the rest of the day was spent being sedentary or doing light PA.^[8]^ According to Parsons et al, older men with a mean age of 78 years with ABI values <1.0 spent a mean of 25 min/d doing MVPA, and the rest of the day was spent being sedentary or doing light PA.^[9]^ Gerage et al reported to have found no association between ABI values and meeting the PA guidelines,^[7]^ whereas an inverse relationship was reported between increasing the duration of PA and ABI values.^[9]^ However, only men were included in these studies,^[8,9]^ and therefore, generalizing these results to women would not be appropriate. Evidence from large population-based studies examining the links between PA levels and PAD severity needs to be elucidated.

Thus, the aim of this study was to characterize PA levels among people with PAD. Moreover, using the ABI, we investigated the extent to which PAD severity explains the differences in PA level among people with PAD. The information gathered from this study is expected to provide health-care providers with information to establish appropriate PA promotion programs and interventions to attenuate the risk of PAD and enhance the quality of life of people with PAD.

## 2. Methods

### 2.1. Study design and participants

In this cross-sectional cohort study, data from the Hispanic Community Health Study/Study of Latinos (HCHS/SOL) for the period 2008 to June 2011 were analyzed. The HCHS/SOL was a multisite community-based cohort study that examined the prevalence of chronic diseases and the associated health factors in a sample of Hispanic/Latino adults aged 18 to 74 years at baseline (2008–2011). The HCHS/SOL recruited 16,415 self-identified Hispanic/Latino people in 4 cities (Bronx, NY; Chicago, IL; Miami, FL; and San Diego, CA). Participants were recruited from randomly selected households. The detailed methods were reported previously.^[8]^ The institutional review boards at each site approved the study, and all procedures adhered to the Declaration of Helsinki. A written informed consent was obtained from the participants for the publication of any associated data as explained in the HCHS/SOL.

**Figure 1.**
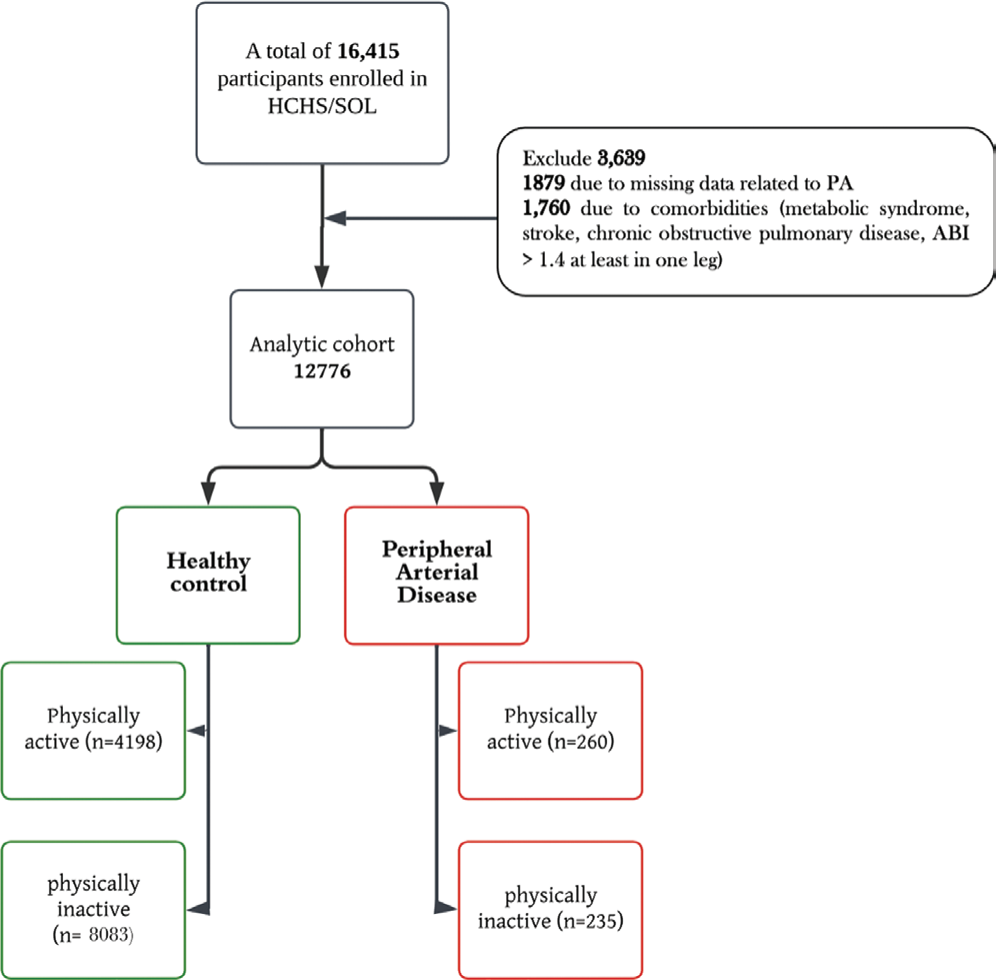
Study sample flow diagram.

In this study, oversampled middle-aged and older adults (45–74 years) who reported having ABI scores from 2008 to 2011 were included. We excluded 1760 participants because they had comorbidities such as chronic obstruction lung disease, stroke, metabolic syndrome, or ABI value of >1.4, and 1879 participants because of missing data related to PA. Therefore, the total primary analytical sample consisted of 12,776 participants. Figure 1 presents the study flow diagram.

## 3. Measures

### 3.1. Demographics and clinical variables

The participants’ demographic information, including age, gender, education, and income, was obtained in the study. The average daily sleep duration during an entire week (5 weekdays and 2 weekend days) was also obtained using self-reported sleep data. At the HCHS/SOL Central Lab at the University of Minnesota Medical Center, participants’ fasting insulin, glycated hemoglobin, blood lipids and lipoprotein levels were measured for exploratory purpose. Dyslipidemia was diagnosed based on the National Cholesterol Education Program/Adult Treatment Panel III clinical guidelines for cholesterol testing. Different patterns of dyslipidemia were defined based on National Cholesterol Education Program/Adult Treatment Panel III guidelines as follows: LDL-C > 130 mg/dL, triglyceride > 200 mg/dL, and low HDL-C (<40 mg/dL for men and <50 mg/dL for women).^[10]^

### 3.2. PAD severity measure

The ABI is a noninvasive measure of PAD (arterial stenosis and stiffness).^[11]^ The standard Doppler procedure using a Nicolet Doppler Elite 100R probe (Natus, Golden, CO) was used to measure the ABI, as described in previous studies.^[12]^ The ABI is considered normal if its value is between 1.0 and 1.4 and abnormal if its value is <1.0 or >1.4.^[9,13]^ An ABI between 0.91 and.99 should be considered borderline PAD. Patients are diagnosed with PAD when their ABI values are ≤0.9 on either the left or right limb.^[3]^ In terms of PAD categories, ABI values 0.7 to <0.90, 0.4 to <0.7, and <0.4 are considered mild, moderate, and severe PAD, respectively. When the ABI is between 0.91 and .99, it is considered borderline, and between 1.0 and 1.4 is considered normal, respectively. An ABI value >1.4 is an indication of the presence of stiff blood vessels, which is not considered PAD.^[3–5]^

### 3.3. PA

The Global Physical Activity (GPA) Questionnaire is self-administered to assess the time spent in a typical week performing combined MVPA during work and leisure time, walking or biking for transportation. Work-related, transportation-related, and leisure-time PA were included in our moderate PA variable. In the vigorous PA variable, only work-related and leisure-time PA were included. During a week, the MVPA and vigorous PA variables were created in accordance with the 2008 Physical Activity Guidelines for Americans. The PA recommendations for the general population, including people with PAD, were classified into 3 categories.^[14]^ The 3 categories for MVPA were as follows: met the guidelines (≥150 min/wk); insufficiently active (>0–< 150 min/wk); and inactive (0 min/wk). The MVPAs performed during the week were in accordance with the 2008 Physical Activity Guidelines for Americans. The variables pertaining to the types of PA were presented as minutes per day. In addition, the overall GPA score was categorized on the basis of the average time spent per week doing any PA. Three PA categories are identified: active “indicated engagement in vigorous activity of at least 20 min/d for 3 or more days or engaged in 5 or more days of moderate-intensity activities or walking for at least 30 min/d which is equivalent to ≥150 min/wk, or 5 or more days of any combination of walking, moderate-intensity, or vigorous-intensity activities totaling at least 600 MET-min/wk”; insufficient defined as not meeting criteria of active but perform PA <150 min/wk and inactive category indicated not engaged any PA.^[14]^

### 3.4. Group classification

In this study, the participants were categorized based on the PA and presence or absence of PAD into 4 groups as follows: healthy control and physically active, healthy control but physically inactive to insufficient, PAD and physically active, and PAD but physically inactive to insufficient.

### 3.5. Data analysis and statistical plan

Baseline characteristics of the study population were compared across 4 different categories of combined PAD and PA. The data were presented as means and standard deviations for normally distributed continuous variables, medians and interquartile range (IQR) for non-normally distributed variables, and frequency and percentages for categorical variables. For categorical demographic characteristics, the Pearson chi-square test was performed to assess the differences in the 4 groups. Further analysis was conducted to compare the differences between PAD – mild and PAD – moderate to severe. In addition, the differences in the continuous variables pertaining to the demographic characteristics were assessed using the Kruskal–Wallis *H* test for the 4 groups (PAD–non-PA, PAD–PA, healthy control and PA, and healthy control PA) and the Mann–Whitney *U* test for the 2 groups (PAD – mild and PAD – moderate to severe). Two main regressions were used in this study: complex-samples multinominal logistic regression modeling was used to investigate the association between overall GPA as an independent variable (inactive, insufficient, and active [reference value]) and PAD severity as a dependent variable (normal [reference], mild, and moderate to severe) after adjusting for age (years), body mass index (BMI), and smoking status. Odds ratios (ORs) and confidence intervals were estimated in the calculation to explain the strength of the association between the dependent and independent variables; complex-samples general linear modeling was used to investigate the association between the type of PA as a dependent variable (continuous values, including work related, transportation related, recreational, and total METs) and PAD severity as an independent variable (continuous ABI values). Both models were adjusted using 2 controlling categories: the first model was used to control for age; and the second model was used to control for the first model and smoking status and BMI. Smoking status was measured as the number of exposure-years multiplied by the average number of cigarettes smoked per day.

The collected data were analyzed using the statistical Package for Social Sciences (SPSS), version 20 (SPSS Inc., Chicago, IL).

## 4. Results

A total of 12,776 participants were included in this study. The participants were divided into 4 groups according to the presence of PAD (i.e., normal or abnormal ABI) and the category of PA (i.e., physically active or inactive). These groups included participants who were physically active with normal ABI ([control] CON–physically active; n = 8083) and physically inactive to insufficient with normal ABI (CON–physically inactive to insufficient; n = 4198), physically active with abnormal ABI (PAD–physically active; n = 260), and physically inactive with abnormal ABI (PAD–physically inactive to insufficient; n = 235). Demographic characteristics of the groups are presented in Table 1. There were significant differences found between the groups in some of the demographic and clinical variables. Investigating the PA among PAD participants showed that the majority of participants with PAD (260; 52.5%) were physically active, a total of 156 (31.6%) participants performed insufficient PA, and 79 (15.9%) participants were physically inactive.

**Table 1:** Sociodemographic and physical characteristics of the study population.

Variables	PAD-physically inactive to insufficient	PAD- physically Active	CON -physically inactive to insufficient	CON- physically active	*P* value
	n = 235	n = 260	n = 4198	n = 8083	
	Mean ± SD	Mean ± SD	Mean ± SD	Mean ± SD	
BMI (kg/m^2^)	30.88 ± 6.80	29.21 ± 5.46	30.40 ± 6.13	29.35 ± 5.73	<.001*
Height (cm)	157.87 ± 8.32	159.85 ± 8.88	160.96 ± 8.81	163.47 ± 9.23	<.001*
Age (yr)	58.80 ± 8.12	57.12 ± 8.14	47.02 ± 13.54	42.88 ± 14	<.001*
Gender, n = 12,771	n = 235	n = 260	n = 4198	n = 8078	<.001*
Female, n (%)	184 (1.44)	164 (1.28)	2850 (22.31)	4086 (31.99)	
Education, n = 12,700	n = 235	n = 256	n = 4195	n = 8014	<.001*
No high school diploma, n (%)	128 (54)	1041 (41)	1698 (40)	2749 (34)	
High school diploma or an equivalent degree, n (%)	107 (45)	152 (59)	2497 (60)	5265 (66)	
Adjusted number of cigarettes per yr	315.73 ± 510.35	435.91 ± 27.58	265.99 ± 4.15	246.70 ± 2.78	<.001*
Dyslipidemia*, n (%)	n = 233	n = 256	n = 4158	n = 7984	.003*
	115 (67)	115 (61)	1611 (39)	3116 (39)	
Sleep duration (h/d)	7.94 ± 1.49	7.80 ± 1.52	8.08 ± 1.46	7.87 ± 1.40	<.001*
Fasting insulin (mU/L)	18.12 ± 48.17	12.82 ± 9.57	14.45 ± 11.91	12.45 ± 10.29	<.001*
HbA1C (mmol/mol)	47.86 ± 16.62	46.20 ± 18.59	41.51 ± 14.21	40.10 ± 13.87	<.001*
MVPA, median (IQR)	29 (60)	615 (1430)	31 (60)	960 (1980)	<.001*

* Dyslipidemia was defined based on National Cholesterol Education Program/Adult Treatment Panel III (NCEP/ATP III) guidelines.

CON = control, BMI = body mass index, HbA1C = glycated hemoglobin, IQR = interquartile range, METS (NCEP-definition) = metabolic syndrome is based on the updated Adult Treat Panel III of the National Cholesterol Education Program (NCEP) guidelines, MVPA = moderate-to-vigorous physical activity, PAD = peripheral arterial disease, SD = standard deviation.

* *P* value ≤.05.

On the basis of PAD severity, Table 2 presents the comparison of the characteristics of the participants with mild PAD and those of the participants with moderate-to-severe PAD, including the significant differences between the 2 groups. A total of 432 participants with PAD in the mild group, 57 participants reported to have moderate PAD, and 6 participants had severe PAD. There were significant differences between people with mild and moderate to severe PAD in all demographic and clinical variables, except BMI, gender, dyslipidemia, and weekday sleep duration. In comparison to mild PAD, the MVPA was significantly low in the moderate to severe PAD (95 [IQR = 0–4300 min/wk] vs 165 [IQR = 0–750 min/wk], *P* = .02).

**Table 2:** Characteristics of individuals with PAD.

Variables	PAD – mild	PAD – moderate to severe	*P* value
	n = 430	n = 63	
	Median (IQR)	Median (IQR)	
BMI (kg/m^2^)	29 (25.55–33.29)	28 (24.42–31.87)	.28*
Height (cm)	158 (152.90–164)	163 (155–169)	.001*
Age (yr)	57 (50.25–63)	62 (57–69)	<.001*
Gender, female, n (%)	220 (51)	31 (49)	.62*
MVPA (min/wk)	165 (0–750)	95 (0–4300)	.02*
Income, n = 430, n (%)			.01*
<$10,000	94 (25)	25 (46)	
$10,001–$20,000	131 (35)	15 (28)	
$20,001–$40,000	111 (29)	9 (17)	
$40,001–$75,000	29 (8)	5 (9)	
>$75,000	11 (3)	0	
Education, n (%)			.002*
No high school diploma	191 (45)	41 (65)	
High school diploma or an equivalent degree	237 (55)	22 (35)	
Number of cigarettes per yr	235.45 (79.53–109.75)	286.47 (61.74–89.30)	.005*
Dyslipidemia, n = 415 yes n (%)	206 (55)	24 (44)	.12*
Sleep duration (h/d)	8 (7–8.5)	8 (7–9)	.24*
Sedentary behavior, yes n (%)	130 (30)	26 (42)	.07*

Values are expressed as mean rank and interquartile value or frequency

BMI = body mass index, IQR = interquartile range, MVPA = moderate-to-vigorous physical activity, PAD = peripheral arterial disease.

* *P* value ≤.05.

Table 3 presents the results of the complex logistic regression on the association of ABI severity with total GPA. Compared with the highly active participants, those who engaged in low PA were twice as likely to have moderate ABI severity and 4 times as likely to have severe ABI after adjustment for age, BMI, and smoking status. Those who engaged in moderate levels of PA had a 37% lower likelihood of having moderate ABI severity. Similarly, those who engaged in moderate PA levels had an 84% lower likelihood of having severe ABI. In addition, a decrease in work-related PA (*β* = −.007; *P* = .05), total METs (*β* = −.001; *P* = .04), and total MVPA (*β* = −.005; *P* = .03) was associated with increased PAD severity after controlling for age, sex, education, smoking status, BMI, and statin (Table 4).

**Table 3:** The associations between level of physical activity and severity of peripheral arterial disease.

ABI categories	Inactive PA vs active PA			Insufficient PA vs Active PA		
	Unadjusted	Model 1*	Model 2†	Unadjusted	Model 1*	Model 2†
	OR (95% CI)	OR (95% CI)	OR (95% CI)	OR (95% CI)	OR (95% CI)	OR (95% CI)
Normal	1	1	1	1	1	1
Mild	0.21 (0.13,0.33)*	0.53 (0.32,0.87)*	0.52 (0.31,0.85)*	0.53 (0.41,0.70)*	0.71 (0.53,0.94)*	0.70 (0.52,0.93)*
Moderate	1.2 (−7.9, 1.80)	1.37 (−7.60, 2.49)	2.38 (1.29, 4.39)*	0.53 (0.22, 1.2)*	0.76 (0.32, 1.85)*	0.63 (0.27, 1.50)*
Severe	8.4 (−3.34, 2.11)	3.38 (1.35, 8.41)*	5.10 (−1.93, 1.34)	0.11 (0.01,0.97)*	0.15 (0.02, 1.28)*	0.16 (0.02, 1.30)*

The dependent variables are the ABI categories, and the reference values are normal ABI and high moderate-to-vigorous physical activity.

ABI = ankle–brachial index, CI = confidence interval, OR = odds ratio, PA = physical activity.

* Adjusted for age, sex, education.

* *P* value ≤.05.

† Adjusted for model 1 covariates, plus body mass index, statin and number of cigarettes per year.

**Table 4:** The associations between type of physical activity and severity of peripheral arterial disease.

Type of PA	ABI severity (continuous variable)							
	Model 1		Model 2 *		Model 3†		Model 4‡	
	*β* Estimate (95% CI)	*P* value	*β* Estimate (95% CI)	*P* value	*β* Estimate (95% CI)	*P* value	*β* (95% CI)	*P* value
Work (min/wk)	−0.008 (−0.018, −0.003)	.02	−0.007 (−0.01–−0.002)	.04	−0.007 (−0.01, −0.002)	.05	−0.007 (−0.02**–**0.002)	.05
Transport (min/wk)	−0.004 (−0.01–0.001)	.22	−0.006 (−0.01–0.001)	.16	−0.005 (−0.01–0.001)	.18	−0.005 (−0.01–0.001)	.48
Recreational (min/wk)	0.000 (−0.01–0.009)	.95	0.005 (−0.008–0.01)	.32	0.006 (−0.008–0.01)	.32	0.006 (−0.008,0.01)	.28
Total metabolic equivalents	−0.001 (−0.002–0.000)	.01	−0.001 (−0.002–−0.001)	.02	−0.001 (−0.002–−0.000)	.03	−0.001 (−0.002–0.000)	.04
Total moderate-to-vigorous PA (min/wk)	−0.005 (−0.01–−0.002)	.01	−0.005 (−0.01–−0.002)	.02	−0.005 (−0.0–0.001)	.02	−0.005 (−0.01–−0.001)	.03

The dependent variable is the type of PA (in min/wk), and the reference value is normal ABI.

ABI = ankle–brachial index, CI = confidence interval, PA = physical activity.

* Adjusted for age, sex, education.

† Adjusted for model 2 covariates, plus smoking status, and body mass index.

‡ Adjusted for model 3 covariates, plus statin.

## 5. Discussion

The overarching aim of this study was to assess PA patterns among individuals with PAD and investigate whether these PA patterns associated with PAD severity. The main findings showed that the likelihood of PAD severity increased for individuals who engaged in insufficient PA. In addition, the PA related to work and total metabolic equivalents per week associated negatively with the severity of PAD based on the ABI scores. The study showed that the majority of participants with PAD (52.5%) were physically active and achieved global PA recommendations (≥150 min/wk), whereas 47.5% were physically inactive to physically insufficient. There was a significant difference in the MVPA level between participants with mild versus moderate to severe PAD. The average amount of engagement in MVPA was higher among participants with mild PAD compared to those with moderate to severe PAD.

Examining PAD severity in association with engagement in MVPA showed a high percentage (16%) of moderate to severe PAD among inactive individuals and only 10% of severe PAD among active individuals. It was found that MVPA patterns were associated with PAD severity and explained the high severity among physically inactive participants with PAD. Furthermore, in this study, the amount of work per week and number of METs consumed per week were influenced by PAD severity, whereas recreational PA was not associated with PAD severity. Health promotion and initiative should be implemented to encourage individuals with PAD to engage in at least some MVPA. Future studies are required to identify appropriate exercise regimens or home-based programs to help participants with severe PAD meeting the current PA recommendations.

### 5.1. PA patterns among participants with PAD

In this study, the participants and healthy control groups were stratified according to their PA patterns. Despite that the 2 groups of healthy participants and those with PAD attained the recommended level of MVPA level, significant differences in the MVPA (min/wk) were still found. Participants with PAD who met the current guidelines for adult MVPA and counted toward the recommended 150 min/wk had significantly lower PA activity compared to those healthy and physically active. When the participants with mild PAD were compared with those with moderate-to-severe PAD, a significant difference in engagement with MVPA was observed. Furthermore, work related PA and total METs were significantly associated with ABI scores. The effects of the lack of PA have been studied previously. Women with symptomatic PAD were found to report less MVPA and more barriers to PA than did men.^[15]^ Contrary to our findings, a previous study reported a decline in PA among female showed that the main barriers to engagement in PA were related to claudication.^[16,17]^ However, a high percentage of our participants with PAD who were physically active reported a lack of claudication symptoms (n = 157; 62%), which could be attributed to our findings. Thus, the participants with PAD who were physically active demonstrated lower ABI values than those with PAD who were physically inactive. These findings concur with previous studies that reported that PA is an important factor in the treatment of participants with intermittent claudication and in encouraging supervised exercise programs to improve functional capacity.^[6,18]^

This study shows that the participants with PAD who met the current PA guidelines displayed mild disease severity than those who did not engage in MVPA of 150 min/wk. The participants with PAD who were physically active had significantly fewer times per week of engagement in MVPA than those without PAD. The findings of this study support a systematic review that provided an association between self-initiated PA and reduced risk of PAD.^[19]^ This finding concurred with a previous study that linked the improvement in atherosclerotic risk factors, such as dyslipidemia, to PA.^[20]^ A previous study showed that exercise programs, especially those that were supervised ones, were more effective in improving walking distance by up to 210 metres after 3 months of intervention among participants with intermittent claudication.^[18]^ It is well documented that PA is useful in the treatment of many established atherosclerotic risk factors such as dyslipidemia, arterial hypertension, obesity, insulin resistance, and glucose intolerance.^[21]^ However, these findings do not prove that exercise impedes the progression of PAD or improves the flow-mediated dilatation of arteries.

The findings of this study distinguished between different types of PA, such as leisure-time, recreational PA, which were reported to lack any significant association with PAD severity. However, the activity that required locomotor and extensive PA levels – such as MVPA – was different between the PAD and healthy groups. These findings may be explained by the disease, as PAD affects legs and could explain our findings.^[22,23]^ The preventive effect against the progression of PAD was reported, and it was reported that walking for >30 minutes at least 3 times per week or to the point of claudication improved pain distance.^[20]^ Future studies are required to determine whether or not the degree of walking impairments alone is associated with low PA levels.

Our results enhance the sparse research linking PA and cardiovascular health in the adult population with PAD. A significant association was found between PA levels and PAD severity. The association between physical inactivity and PAD was robust after controlling for sex, age, education, number of cigarettes per year, BMI, and statins. The inactive participants with PAD had a significantly higher OR of developing moderate to severe PAD than the physically active participants with PAD. This is consistent with previous research that examined the association between PA and PAD and showed a significantly lower OR of developing PAD in participants who engaged in PA.^[24]^ Our finding that PAD severity is favorably related to MVPA performed, but not recreational or transportation-related PA, embraces the evidence of a link between the type of activity and its intensity and degree of severity in adults with PAD. There is a lack of clear evidence for the association between PA and incident PAD, as prospective cohort studies are required to detect the association between PA levels and PAD severity.

We acknowledge that this study has several limitations. This is a cross-sectional study that provides information on the co-occurrence of low PA and PAD. However, our findings do not imply that the causation of the progression of PAD is the impact of PA. Furthermore, the presence of claudication in participants with PAD of various severity levels would more likely affect engagement in PA. However, our analysis ruled out any differences in the presence of claudication between the groups. Thus, the small sample of participants with PAD precluded the generalization of this study and resulted in a lack of statistical power to precisely estimate the association between PAD severity and PA patterns. The recommended MVPA level was measured on the basis of the 2008 Physical Activity Guidelines for Americans.^[14]^ The self-administered questionnaire used to evaluate PA in the study had self-reported bias, measurement errors, and limited measurement of the context in which PA was performed. Future studies are required to objectively measure PA data using accelerometers. Although a wide range of confounding factors in the regression models were adjusted for in this study, other factors that might have been present must be taken into consideration. The results should be carefully extrapolated to other patient groups.

## 6. Conclusions

This study showed that the likelihood of PAD severity might be increased for individuals who engaged in low PA. The study findings highlight the association between PA and PAD severity, although better-powered studies are needed to study the physiological mechanism underlying this relationship. In addition, it might be better to measure the ABI and PA level during regular clinic visits to overcome any possible future complications related to severe PAD.

## Acknowledgment

We would like to express our gratitude to Princess Nourah bint Abdulrahman University for supporting this project through Princess Nourah bint Abdulrahman University Researchers Supporting Project number (PNURSP2022R286), Princess Nourah bint Abdulrahman University, Riyadh, Saudi Arabia.

## Author contributions

MA, MMA contributed to the conception and analysis of the study. MA, AA, and MMA were involved in preparation of the manuscript and reviewed the manuscript for important intellectual content. MMA conducted that statistical analysis of the data.

**Conceptualization:** Monira I. Aldhahi, Mohammed M. Alshehri.

**Data curation:** Monira I. Aldhahi, Mohammed M. Alshehri.

**Formal analysis:** Monira I. Aldhahi, Mohammed M. Alshehri.

**Funding acquisition:** Monira I. Aldhahi.

**Investigation:** Monira I. Aldhahi, Abdulfattah S. Alqahtani.

**Methodology:** Monira I. Aldhahi, Mohammed M. Alshehri, Abdulfattah S. Alqahtani.

**Project administration:** Monira I. Aldhahi.

**Resources:** Monira I. Aldhahi.

**Supervision:** Monira I. Aldhahi.

**Visualization:** Monira I. Aldhahi.

**Writing – original draft:** Monira I. Aldhahi, Mohammed M. Alshehri, Abdulfattah S. Alqahtani.

**Writing – review & editing:** Monira I. Aldhahi, Mohammed M. Alshehri, Abdulfattah S. Alqahtani.
